# miRNA Expression Profile and Effect of Wenxin Granule in Rats with Ligation-Induced Myocardial Infarction

**DOI:** 10.1155/2017/2175871

**Published:** 2017-08-15

**Authors:** Aiming Wu, Lixia Lou, Jianying Zhai, Dongmei Zhang, Limin Chai, Bo Nie, Haiyan Zhu, Yonghong Gao, Hongcai Shang, Mingjing Zhao

**Affiliations:** ^1^Key Laboratory of Chinese Internal Medicine of Ministry of Education and Beijing, Dongzhimen Hospital Affiliated to Beijing University of Chinese Medicine, Beijing 100700, China; ^2^National Engineering Research Center for R&D of TCM Multi-ingredient Drugs, Beijing 100079, China; ^3^Beijing University of Chinese Medicine Institute for Cardiovascular Disease, Dongzhimen Hospital Affiliated to Beijing University of Chinese Medicine, Beijing 100700, China

## Abstract

Wenxin Granule (WXKL) is a traditional Chinese medicine used for treatment of myocardial infarction (MI) and arrhythmias. However, the genomic pathological mechanisms of MI and mechanisms of WXKL are largely unknown. This study aims to investigate a comprehensive miRNA expression profile, and the predicted correlation pathways to be targeted by differentially expressed miRNAs in MI, and mechanisms of WXKL from a gene level. MI rat model was established by a coronary artery ligation surgery. miRNA expression microarrays were performed and the data were deposited in Gene Expression Omnibus (GEO number GSE95855). And, pathway analysis was performed by using the DIANA-miRPath v3.0 online tool. The expressions of miR-1, miR-133, Cx43, and Cx45 were detected by quantitative real-time PCR. It was found that 35 differentially expressed miRNAs and 23 predicted pathways, including miR-1, miR-133, and gap junction pathway, are involved in the pathogenesis of MI. And, WXKL increased the expressions of miR-1 and miR-133, while also increased the mRNA levels of Cx43 and Cx45, and, especially, recovered the Cx43/Cx45 ratio near to normal level. The results suggest that regulatory effects on miR-1, miR-133, Cx43, and Cx45 might be a possible mechanism of WXKL in the treatment of MI at the gene level.

## 1. Introduction

Myocardial infarction (MI) is a serious cardiovascular disease that threatens human health. It remains one of the predominant causes of morbidity and mortality even though great efforts have been made to manage it [1]. In China, the mortality from MI is increasing attributable to population aging [2]. Arrhythmia, especially ventricular fibrillation, is one of the leading causes of death in patients with MI. And, in more than half of sudden death cases, ventricular fibrillation appears as the first symptom during MI [3]. Therefore, further research of the pathological mechanism and finding suitable agents are very important to prevent potential lethal arrhythmia following MI.

In recent years, natural product medicine, such as traditional Chinese medicine, has drawn great attention from people around the world and has been playing important roles in the prevention and treatment of cardiovascular diseases [4–6]. A traditional Chinese medicine named Wenxin Granule (WXKL) has been reported to prevent MI and arrhythmias [7–9]. However, the pharmacological mechanisms of WXKL at the genomic level in MI are largely unknown.

MicroRNAs (miRNAs) have been demonstrated as critical factors involved in various cardiovascular diseases including arrhythmias following MI [10, 11]. As well known, posttranscriptional regulation is a pivotal and precise regulatory mechanism that plays an important role in the process of gene expression. miRNAs play central roles in posttranscriptional regulation processes, leading to the inhibition of one or more posttranscriptional target genes silencing and then regulating the corresponding pathways. miRNAs are a class of approximately 20–22 nucleotide long, endogenous noncoding RNAs. The functional strand of mature miRNA could regulate protein expression at the posttranscriptional level by binding to the 3′ untranslated regions of the target mRNA [12]. Since the initial discovery of miRNAs in 1993 (Lee et al., 1993), it has so far registered over 1800 miRNAs, which target about 60% of human genes, in the miRBase database (http://www.mirbase.org/) [13]. Recently, much evidence has indicated that miRNAs play an important role in the pathogenesis of cardiovascular disease, including coronary artery disease, myocardial infarction, atherosclerosis, arrhythmias, and heart failure, by involving in specific signaling pathways [14–17]. However, a comprehensive miRNA expression profile, especially the pathways predicted to be targeted by differentially expressed miRNAs in ligation-induced MI rats, is still unclear.

Recent progress in miRNA expression microarray has enabled the use of the high throughput technologies to obtain an in-depth understanding of the pathological mechanisms of MI and pharmacological mechanisms of natural product medicine at the genomic level. In present study, a MI rat model was established by a direct coronary artery ligation surgery method and confirmed by electrocardiographic (ECG) and histopathological methods. Then, left ventricular tissues from 3 MI rats and 3 non-MI rats (control) were collected for miRNA expression microarray analyses. To assess the pathways predicted to be targeted, pathway analysis of differentially expressed miRNAs was performed by using the DIANA-miRPath v3.0 tool. The relative expressions of miR-1 and miR-133 were validated by quantitative real-time PCR, and the possible effects of WXKL were observed at the same time. Additionally, the effect of Wenxin Granule on Cx43 and Cx45, those involved in gap junction pathway, was also observed in the present study.

## 2. Materials and Methods

### 2.1. Animals

A total of 50 male Sprague-Dawley (SD) rats, weighted 200 ± 20 g, were acquired from Beijing Vital River Laboratory Animal Technology Co. Ltd. (License number SCXK (Beijing) 2012-0001).

### 2.2. Drugs

Wenxin Granule (SFDA Approval number Z10950026) was manufactured by Shandong Buchang Pharmaceuticals Co. Ltd., Xi'An, China. Captopril tablets (SFDA Approval number H31022986) were manufactured by Sino-American Shanghai Squibb Pharmaceuticals Ltd., Shanghai, China.

### 2.3. The MI Rat Model Preparation

The model was established by a direct coronary artery ligation surgery method as described previously [18]. Before surgery, the rats were anaesthetized with 1% pentobarbital sodium (50 mg/kg) intraperitoneally, and a twelve-lead electrocardiogram (ECG) was performed preoperatively. After left thoracotomy, the left anterior descending coronary artery was ligated directly at the location between the pulmonary cone and the left atrial appendage under its origin 2-3 mm in all groups except the control. Additionally, a twelve-lead ECG was performed postoperatively the day after the surgery. Whether the surgery was successful can be judged by Q wave in postoperative ECG, compared with preoperative ECG. In the experimental period, the total mortality rate was 20% to 30%. The main causes of death were lethal arrhythmias, respiratory failure, and acute pump failure.

### 2.4. Design and Allocation

This protocol was approved by the Standing Committee on Animals at Dongzhimen Hospital Affiliated to Beijing University of Chinese Medicine. All animals used in this study received humane care in compliance with the National Institutes of Health Guide for the standards for ethical treatment of laboratory animals. And, efforts were made to minimize the number of animals used. The MI rats with a successful coronary artery ligation surgery were assigned randomly into the model group, the captopril group, and the WXKL group. Meanwhile, the rats without coronary artery ligation were assigned to the control group, with 9 rats in each group. The day after the surgeries, treatments were administered to the rats intragastrically for 4 weeks. All drugs were ground and mixed with distilled water before administration. The captopril group was given with a dosage of 2.2 mg/kg of captopril tablets. The WXKL group was given with a dosage of 2.7 g/kg of WXKL. The sham group and the model groups received the same volume of distilled water via oral gavage. After 4 weeks of treatments, all rats were anaesthetized and dissected to isolate the heart for the subsequent experiments.

### 2.5. Masson Trichrome Staining

Masson trichrome staining was performed with tissue that was fixed in 4% paraformaldehyde and embedded in paraffin. The tissue was cut into 4 *μ*m sections using a paraffin slicer. The main procedures performed are as follows: deparaffinize and rehydrate through xylene and a series of ethanol washes (100, 95, 90, 80, and 70% alcohol), stain in Biebrich scarlet-acid fuchsin solution for 15 minutes, wash in distilled water, differentiate in phosphomolybdic-phosphotungstic acid solution for 5 minutes, transfer sections directly to brilliant green solution and stain for 10 minutes. rinse briefly in distilled water and differentiate in 1% acetic acid solution for 1 minute, wash in distilled water, dehydrate very quickly through 95% alcohol, 100% alcohol, and clear in xylene, and mount with resinous mounting medium.

### 2.6. Microarray Hybridization

RNA was extracted by mirVana™ RNA Isolation Kit (Applied Biosystem, Foster, CA, USA) following the manufacturer's instructions. Cyanine-3- (Cy3-) labeled cRNA was prepared from 0.2 *μ*g RNA using the One-Color Low RNA Input Linear Amplification PLUS kit (Agilent Technologies, Santa Clara, CA) according to the manufacturer's instructions, followed by RNeasy column purification (QIAGEN, Valencia, CA). Dye incorporation and cRNA yield were checked with the NanoDrop ND-1000 Spectrophotometer. Then, 0.6 *μ*g of Cy3-labelled cRNA (specific activity > 10.0 pmol Cy3/*μ*g cRNA) was fragmented at 60°C for 30 minutes in a reaction volume of 22.5 *μ*l containing 1x Agilent fragmentation buffer and 2x Agilent blocking agent following the manufacturers' instructions. On completion of the fragmentation reaction, 22.5 *μ*l of 2x Agilent hybridization buffer was added to the fragmentation mixture and hybridized to Agilent Rat miRNA (8^∗^15K, Design ID: 070154) for 17 hours at 65°C in a rotating Agilent hybridization oven. After hybridization, microarrays were washed 1 minute at room temperature with GE Wash Buffer 1 (Agilent) and 1 minute with 37°C GE Wash buffer 2 (Agilent), then dried immediately by brief centrifugation. Slides were scanned immediately after washing on the Agilent DNA microarray scanner (G2505C) using one color scan setting for 4 × 180 k array slides (scan area 61 × 21.6 mm, scan resolution 3 um; dye channel is set to Green and Green PMT is set to 100%). The microarray data discussed in this study have been deposited in the National Center for Biotechnology Information (NCBI) Gene Expression Omnibus (GEO) and are accessible through (GEO) Series accession number GSE95855 (https://www.ncbi.nlm.nih.gov/geo/query/acc.cgi?&acc=GSE95855).

### 2.7. Bioinformatic Analysis

The scanned images were analyzed with Feature Extraction Software 10.7.1.1 (Agilent Technologies) using default parameters to obtain background subtracted and spatially detrended processed signal intensities as the raw data. Raw data were normalized in quantile algorithm with Genespring 13.0 (Agilent Technologies). The probes that at least 100.0 percent of samples in any 1 condition out of 2 conditions have flags in "Detected" were maintained for further data analysis. Differentially expressed miRNAs were then identified through fold change as well as *P* value calculated using *t*-test. The threshold set for up- and downregulated genes was a fold change ≥ 2.0 and a *P* value ≤ 0.1. Hierarchical clustering was performed to show the distinguishable miRNA expression pattern among samples. Kyoto Encyclopedia of Genes and Genomes (KEGG) pathway enrichment analyses were performed by using the online DIANA-miRPath v3.0 tool (http://www.microrna.gr/miRPathv3) to identify the main functions of the differentially expressed miRNAs [19].

### 2.8. Real-Time Quantitative RT-PCR

Quantification was performed with a two-step reaction process: reverse transcription (RT) and PCR. MicroRNA was reversely transcribed using TaqMan® microRNA Reverse Transcription Kit (Catalog number 4366596, Applied Biosystems, Foster, CA, USA) and then used for quantitative real-time PCR using FastStart Universal SYBR Green Master (Rox) (Catalog number 04913914001, Roche, Swiss) according to the manufacturer's instructions. U6 was used as internal controls. The microRNA-specific primer sequences were performed using TaqMan microRNA Assays (Catalog number 4427975, Applied Biosystems). The mRNA was reversely transcribed using Thermo Scientific RevertAid First Strand cDNA Synthesis Kit (Catalog number #K1622, Thermo Fisher Scientific Inc., USA). The mRNA quantitative real-time PCR was performed using SYBR Green PCR Master Mix (Catalog number 4309155, Applied Biosystems) according to the manufacturer's instructions. GAPDH was used as internal controls. The mRNA-specific primer sequences were designed and synthesized as follows: Cx43 forward: 5′-CAACAACCTGGCTGCGAAAA-3′; reverse: 5′-ACCTTGCCGTGCTCTTCAAT-3′. Cx45 forward: 5′-GGGCTCTGGAAGAAACGGAA-3′; reverse: 5′-ATGCTTGGGTTTTGGTTGGC-3′. GAPDH forward: 5′-AGTTCAACGGCACAGTCAAG-3′; reverse: 5′-TACTCAGCACCAGCATCACC-3′. The expression levels of microRNAs were normalized to U6. The expression levels of mRNAs were normalized to GAPDH. And, the expression levels of microRNAs and mRNAs were calculated using the 2^−ΔΔCt^ method [20].

### 2.9. Statistical Analysis

SPSS software package 13.0 for windows was used for data analysis. Continuous variables were expressed as mean ± standard deviation (SD). Statistical analysis was carried out on three or more groups by one-way analysis of variance (ANOVA) and LSD (Fisher's least significant difference) test. A value of *P* < 0.05 was considered statistically significant.

## 3. Results

### 3.1. Identification of the MI Rat Model

**Figure 1:**
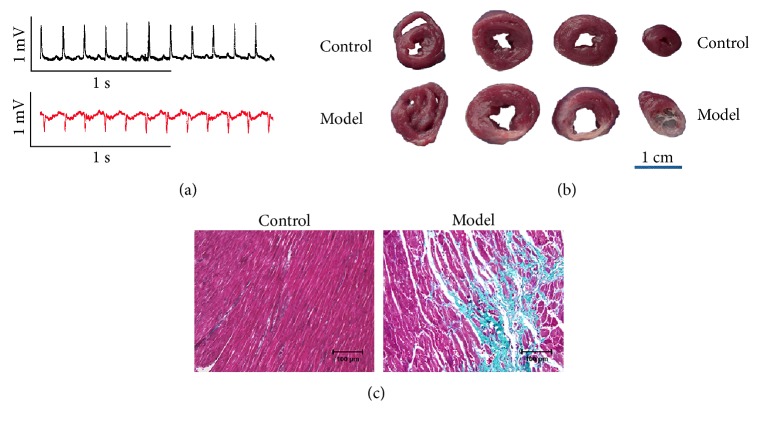
Electrocardiogram (ECG), heart anatomical samples, and Masson trichrome staining from normal and MI rats. (a) Typical ECG recordings. (b) Heart anatomical samples. Scale bars = 1 cm. (c) Masson trichrome staining. Green staining indicates myocardial fibrosis. Scale bars = 100 *μ*m.

We first determined whether the coronary artery ligation surgery was successful in the present study. As shown in Figure 1(a), the ECG of the model group exhibited pathological Q waves. As shown in Figure 1(b), anatomical samples of the heart could be observed with significant MI scarring in the model group. As shown in Figure 1(c), local tissue fibrosis could be observed in the model group by Masson trichrome staining. The above results confirmed the MI model reliability that we used in the present study.

### 3.2. miRNA Expression Signature and Hierarchical Clustering Analysis of MI Rat Model

**Figure 2:**
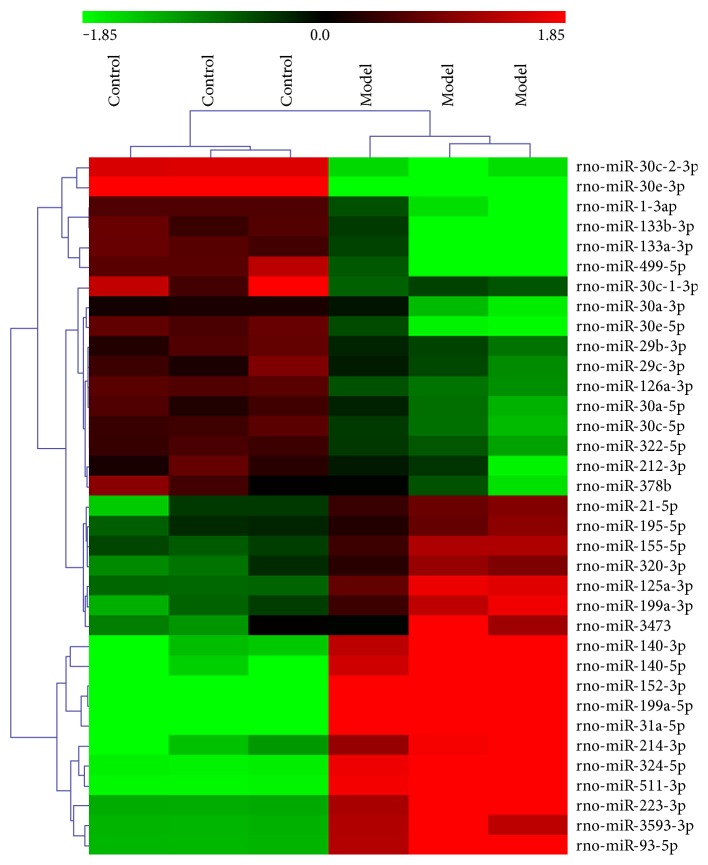
Heatmap of miRNA expression microarray from left ventricular tissue samples of MI rats (model group) and non-MI rats (control group). At 4 weeks after the coronary artery occlusion surgery, heatmap of 35 differentially expressed miRNAs between MI and non-MI rats identified by microarray. The miRNA clustering tree is hierarchically clustered on the left, and the sample clustering tree is hierarchically clustered on the top. The samples are clustering significantly into two groups, the control (non-MI rats) and model (MI rats). The color scale of the miRNA represented in the corresponding row shows the relative expression level of miRNAs; green indicates downregulation, while red indicates upregulation.

At 4 weeks after the coronary artery occlusion surgery, miRNA expression profile was tested between the control and MI rat models. As shown in Figure 2, a total of 35 differentially expressed (more than a twofold change) miRNAs were identified. Compared with the control group, 17 miRNAs were downregulated in the model group as shown in the upper portion of Figure 2. And the other 18 miRNAs were upregulated as shown in the lower portion of Figure 2. Thereafter, hierarchical clustering analysis illustrated that differentially expressed miRNAs could distinguish control and model samples apparently as shown in the sample clustering tree (on the top of Figure 2).

### 3.3. Target Prediction of Differentially Expressed miRNAs

**Figure 3:**
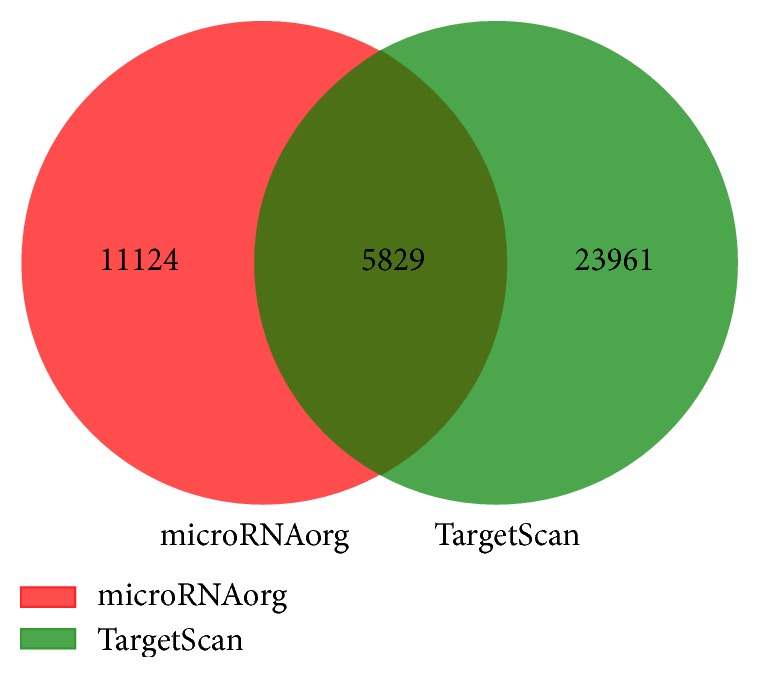
Target genes predicted based on TargetScan database and microRNAorg database.

Target genes of differentially expressed miRNAs were the intersection predicted with TargetScan and microRNAorg databases. As shown in Figure 3, a total of 5829 potential target genes were predicted in the common set of the two databases.

### 3.4. Pathway Analysis of Differentially Expressed miRNAs

**Figure 4:**
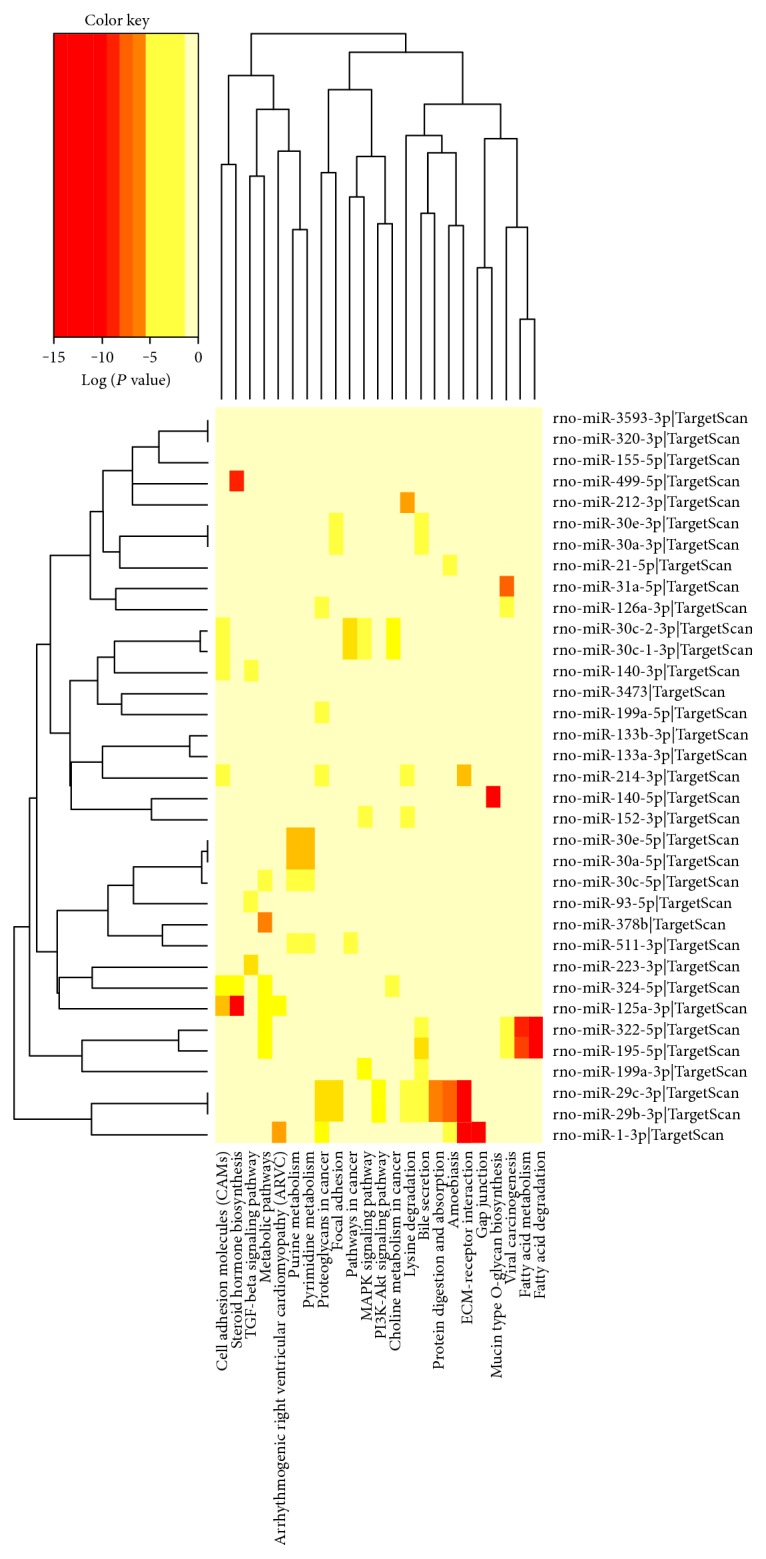
KEGG annotations of differentially expressed miRNAs from left ventricular tissue samples of MI rats (model group) and non-MI rats (control group). The miRNA versus pathway heatmap was created directly from the DIANA-miRPath v3.0 online tool. The heatmap depicts the level of enrichment in pathways of 35 differentially expressed miRNAs between MI and non-MI rats identified by microarray. There were 23 predicted pathways integrating with 35 differentially expressed miRNAs in the heatmap. The pathway clustering tree is shown on the top, and the legend on the bottom indicates the pathway represented in the corresponding column. The miRNA clustering tree is shown on the left, and the legend on the right indicates the miRNA represented in the corresponding row. The color scale shown on the upper left corner illustrates the predicted correlation degree of pathways with the miRNAs (shown as log *P* value).

To assess the pathways predicted to be targeted, pathway analysis of differentially expressed miRNAs was performed by using the DIANA-miRPath v3.0 tool. As shown in Figure 4, the 23 pathways were predicted to be related to the 35 former differentially expressed miRNAs. ECM-receptor interaction, fatty acid metabolism, TGF-beta signaling, and gap junction pathway were involved in these predicted pathways.

### 3.5. Relative Expressions of miR-1 and miR-133

**Figure 5:**
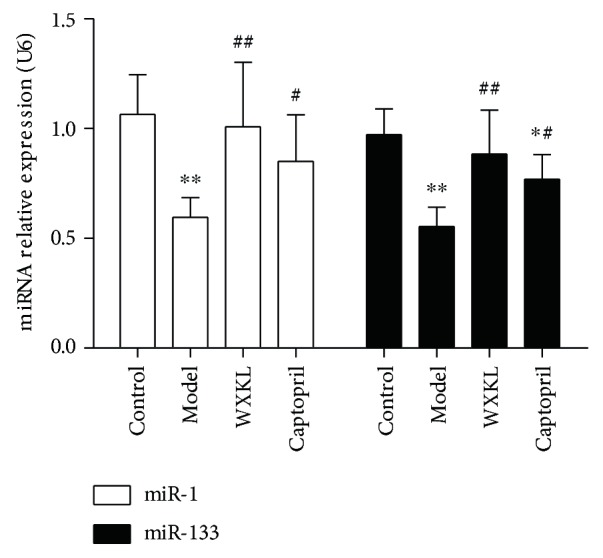
Relative expressions of miR-1 and miR-133. Quantitative real-time PCR was performed to detect the relative expressions of miR-1 and miR-133. Values are expressed as the mean ± SD. ^∗^*P* < 0.05, ^∗∗^*P* < 0.01, versus the control group. ^#^*P* < 0.05, ^##^*P* < 0.01, versus the model group.

The relative expressions of miR-1 and miR-133 were validated by quantitative real-time PCR, and the possible effects of WXKL were observed at the same time. The relative expression of miRNAs was normalized against that of the U6 endogenous control. As shown in Figure 5, the relative expression of miR-1 decreased in the model group compared with the control group (*P* < 0.01). Compared with the control group, the relative expression of miR-133 decreased in the model and the captopril groups (*P* < 0.01 and *P* < 0.05, resp.). Compared with the model group, the relative expressions of miR-1 and miR-133 increased in the WXKL and the captopril groups (*P* < 0.01 and *P* < 0.05, resp.).

### 3.6. Pathway Analysis of Differentially Expressed Cardiac-Specific miRNAs

**Figure 6:**
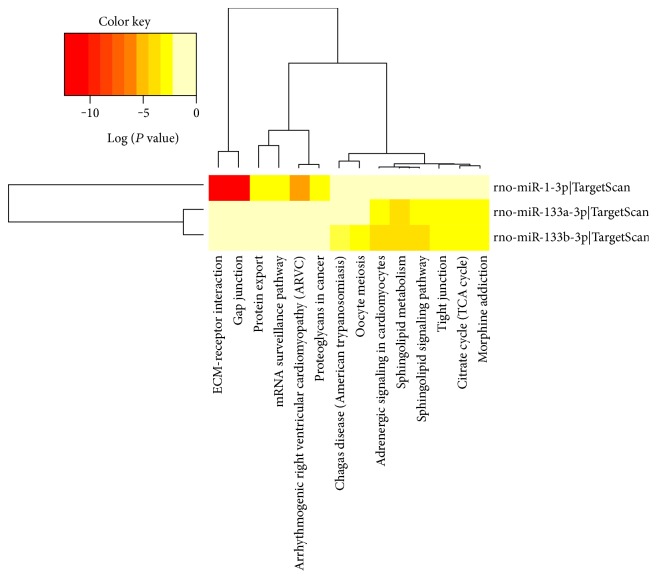
KEGG annotations of differentially expressed cardiac-specific miRNAs from left ventricular tissue samples of MI rats (model group) and non-MI rats (control group). The miRNA versus pathway heatmap was created directly from the DIANA-miRPath v3.0 online tool. The heatmap depicts the level of enrichment in pathways of 3 differentially expressed cardiac-specific miRNAs. There were 14 predicted pathways integrating with 3 differentially expressed cardiac-specific miRNAs in the heatmap. The pathway clustering tree is shown on the top, and the legend on the bottom indicates the pathway represented in the corresponding column. The miRNA clustering tree is shown on the left, and the legend on the right indicates the miRNA represented in the corresponding row. The color scale shown on the upper left corner illustrates the predicted correlation degree of pathways with the miRNAs (shown as log *P* value).

MiR-1 and miR-133 are muscle-enriched miRNAs, and they are abundant in the heart. To further assess the pathways predicted to be targeted, pathway analysis of differentially expressed cardiac-specific miRNAs was performed by using the DIANA-miRPath v3.0 tool. As shown in Figure 6, the 14 pathways were predicted to be related to the 3 differentially expressed miR-1 and miR-133 family members. ECM-receptor interaction and gap junction pathway were the predicted highest correlation.

### 3.7. Relative mRNA Levels of Connexin 43 (Cx43) and Connexin 45 (Cx45)

**Figure 7:**
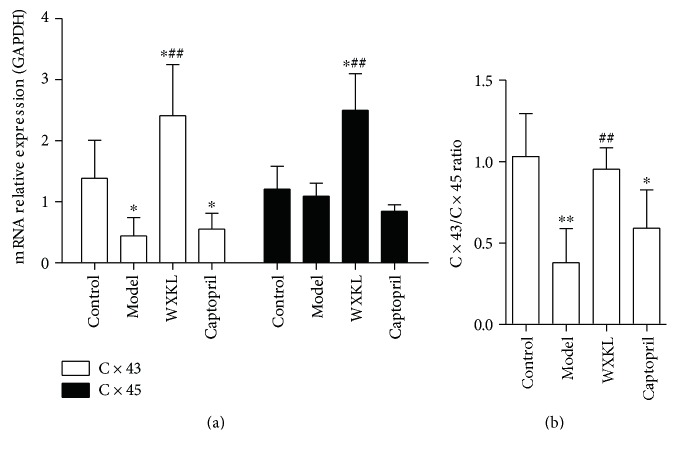
Relative mRNA levels of connexin 43 (Cx43) and connexin 45 (Cx45). (a) The relative mRNA levels of Cx43 and Cx45. (b) The Cx43/Cx45 ratio. Values are expressed as the mean ± SD. ^∗^*P* < 0.05, ^∗∗^*P* < 0.01, versus the control group. ^##^*P* < 0.01, versus the model group.

The mRNA levels of Cx43 and Cx45 are the important factors involved in gap junction pathway. The relative mRNA levels of Cx43 and Cx45 were detected by quantitative real-time PCR, and the possible effects of WXKL were observed at the same time. As shown in Figure 7(a), the relative mRNA levels of Cx43 decreased in the model and the captopril groups compared with the control group (*P* < 0.05). Compared with the model and the control groups, the relative mRNA levels of Cx43 and Cx45 increased in the WXKL group (*P* < 0.01 and *P* < 0.05, resp.). As shown in Figure 7(b), the Cx43/Cx45 ratio decreased in the model and the captopril groups compared with the control group (*P* < 0.01 and *P* < 0.05, resp.). Compared with the model group, the Cx43/Cx45 ratio increased in the WXKL group (*P* < 0.01).

## 4. Discussion

miRNAs are approximately 20–22 nucleotide long, noncoding, endogenous single-stranded RNAs [21]. Studies have confirmed that miRNAs are indeed implicated in the pathogenesis of MI by involving in specific signaling pathways [22–24]. However, a comprehensive miRNA expression profile, especially the pathways predicted to be targeted by differentially expressed miRNAs in MI, is largely unknown.

In the present study, a MI rat model was established by ligation of the left anterior descending coronary artery. This method is the most commonly used experimental model to induce MI in rodents. At 4 weeks after the coronary artery occlusion surgery, the pathological Q waves, scar tissue, and myocardial fibrosis could be observed in the model group. Those results confirmed the MI model reliability in the present study. In order to understand the pathological mechanisms of MI better, the complete miRNA expression state of MI rats and non-MI rats was examined using Agilent Rat miRNA microarray (8^∗^15K, Design ID: 070154) of a total of 758 miRNA probes. The miRNA microarray makes it possible to measure the expression levels of almost all the known rat miRNAs and therefore facilitates the identification of miRNAs and the targeted pathways that are related to MI. In this study, a total 35 differentially expressed miRNAs were identified with more than a twofold change. In the model group, 17 miRNAs were downregulated, including miR-1, miR-133, miR-29, miR-126, miR-212, miR-499, miR-322, miR-378, and miR-30 family members, whereas the other 18 miRNAs were upregulated, including miR-21, miR-195, miR-155, miR-320, miR-125, miR-199, miR-214, miR-324, and miR-140 family members. Among these differentially expressed miRNAs, miR-1, miR-133, miR-29, miR-126, miR-499, miR-30, miR-21, miR-195, miR-155, miR-199, miR-214, and miR-140 have been reported to be related to MI [25–36], while the other miRNAs have not been reported directly in MI. As well known, miRNAs are likely to participate in numerous disease initiation and development by regulating specific target genes. Each miRNA can regulate up to dozens of mRNAs, while multiple miRNAs have been also shown to collaborate in targeting a specific mRNA [37]. Based on the findings of this study, the total 35 differentially expressed miRNAs were identified to target 5829 mRNAs in the intersection predicted with TargetScan and microRNAorg databases. Consequently, many signaling pathways composed of numerous mRNAs are involved in the pathogenesis of MI. But, the numerous miRNAs and target mRNAs pose a significant bottleneck to the elucidation of their functional impact. Fortunately, the DIANA-miRPath v3.0 online tool offers an extensive array of fundamental tools that enable the functional annotation of one or more miRNAs [19]. To assess the pathways predicted to be targeted, pathway analysis of differentially expressed miRNAs was performed by using the DIANA-miRPath v3.0 tool in this study. Pathway analysis showed that many pathways are involved in MI, including ECM-receptor interaction, TGF-beta signaling, fatty acid metabolism, and gap junction pathway. Although extracellular matrix (ECM) plays an important role in the maintenance of myocardial tissue structure integrity and cardiac pump function, excessive ECM remodeling may lead to ventricular diastolic and systolic dysfunctions and ultimately contributes to heart failure [38, 39]. Notably, extracellular matrix synthesis and degradation are closely related to TGF-beta signaling pathway [40]. It is well known that fatty acid and glucose serve a wide variety of functions in the heart. Reportedly, fatty acid dysmetabolism is an important factor which contributes to post-MI cardiac dysfunction and remodeling [41]. Additionally, gap junction pathway is recognized as one of the substrates in susceptibility to post-MI arrhythmias [42].

The above results indicate that the miRNAs and specific signaling pathways might be potential therapeutic targets for treatment of MI. The present study is interested in miR-1 and miR-133, two muscle-enriched miRNAs, and they were chosen for further validation by the quantitative real-time PCR, and the possible effects of WXKL were observed at the same time. Additionally, the effect of WXKL on Cx43 and Cx45, those involved in gap junction pathway, was also detected in the present study. The results showed that the expressions of miR-1 and miR-133 were consistent with the microarray data. And WXKL increased the expressions of miR-1 and miR-133 significantly. MiR-1 and miR-133 have been regarded as key factors involved in cardiac development and cardiovascular disease. Reportedly, mice lacking miR-1-2 develop ventricular septal abnormalities and cardiac rhythm disturbances [43]. While the deficiency of miR-133a leads to myocardial matrix remodeling and progress of heart failure [44, 45]. Further pathway analysis indicated that gap junction pathway was the predicted closely correlation pathway to be targeted by miR-1 and miR-133. Notably, Cx43 and Cx45 are the important factors involved in gap junction pathway, and they are indeed required to maintain cardiac rhythms [46]. One study has revealed that multiple miRNA binding sequences exist in 3′-untranslated regions of Cx43 and Cx45 genes [47]. It has been reported that Cx43 is a miR-1 and miR-133 target [48, 49], but Cx45 has not been reported yet. Both Cx43 and Cx45 are the principal connexins which are expressed in the left ventricle [50]. The change of Cx43/Cx45 ratio has been demonstrated to increase susceptibility to cardiac rhythmicity and reduce gap-junctional intercellular communication [46, 51–53]. In the present study, we demonstrated that WXKL increased the relative mRNA levels of Cx43 and Cx45, and, especially, recovered the Cx43/Cx45 ratio near to normal level. Some studies have confirmed that WXKL is an effective alternative medicine that can improve myocardial ischemia, enhance cardiac function, relieve ventricular remodeling, and reduce the occurrence of arrhythmia [7, 9, 18, 54]. The observed beneficial effects of WXKL in the two connexins can be partly attributed to the above cardioprotective effects. The above findings provide a possible pharmacological mechanism of WXKL in the treatment of MI at the genomic level.

## 5. Conclusions

Complex changes of miRNAs and related pathways, including miR-1, miR-133, and gap junction pathway, are involved in the pathogenesis of MI. Regulatory effects on miR-1, miR-133, Cx43, and Cx45 might be a possible pharmacological mechanism of WXKL in the treatment of MI at the gene level.
